# The Clinical Decision Support System AMPEL for Laboratory Diagnostics: Implementation and Technical Evaluation

**DOI:** 10.2196/20407

**Published:** 2021-06-03

**Authors:** Maria Beatriz Walter Costa, Mark Wernsdorfer, Alexander Kehrer, Markus Voigt, Carina Cundius, Martin Federbusch, Felix Eckelt, Johannes Remmler, Maria Schmidt, Sarah Pehnke, Christiane Gärtner, Markus Wehner, Berend Isermann, Heike Richter, Jörg Telle, Thorsten Kaiser

**Affiliations:** 1 Institute of Laboratory Medicine, Clinical Chemistry und Molecular Diagnostics University of Leipzig Medical Center Leipzig Germany; 2 Faculty of Medicine University of Leipzig Leipzig Germany; 3 Xantas AG Leipzig Germany; 4 Information Management University of Leipzig Medical Center Leipzig Germany; 5 Muldental Clinics GmbH Non-Profit Company Hospital Grimma and Wurzen Grimma Germany

**Keywords:** clinical decision support system (CDSS), laboratory medicine, digital health, reactive software agent, computational architecture

## Abstract

**Background:**

Laboratory results are of central importance for clinical decision making. The time span between availability and review of results by clinicians is crucial to patient care. Clinical decision support systems (CDSS) are computational tools that can identify critical values automatically and help decrease treatment delay.

**Objective:**

With this work, we aimed to implement and evaluate a CDSS that supports health care professionals and improves patient safety. In addition to our experiences, we also describe its main components in a general manner to make it applicable to a wide range of medical institutions and to empower colleagues to implement a similar system in their facilities.

**Methods:**

Technical requirements must be taken into account before implementing a CDSS that performs laboratory diagnostics (labCDSS). These can be planned within the functional components of a reactive software agent, a computational framework for such a CDSS.

**Results:**

We present AMPEL (Analysis and Reporting System for the Improvement of Patient Safety through Real-Time Integration of Laboratory Findings), a labCDSS that notifies health care professionals if a life-threatening medical condition is detected. We developed and implemented AMPEL at a university hospital and regional hospitals in Germany (University of Leipzig Medical Center and the Muldental Clinics in Grimma and Wurzen). It currently runs 5 different algorithms in parallel: hypokalemia, hypercalcemia, hyponatremia, hyperlactatemia, and acute kidney injury.

**Conclusions:**

AMPEL enables continuous surveillance of patients. The system is constantly being evaluated and extended and has the capacity for many more algorithms. We hope to encourage colleagues from other institutions to design and implement similar CDSS using the theory, specifications, and experiences described in this work.

## Introduction

### Background

Clinical decision support systems (CDSS) are computational or technological systems designed to attend specific demands in health care [1-3]. CDSS aim to assist physicians and nurses in making better informed clinical decisions and ultimately improve patient safety [4]. Importantly, the implementation of a CDSS in a health care environment requires the integration of the system into the pre-existing dataflow, computational infrastructure, and clinical procedures [3,5].

Commonly, hospitals use enterprise software in their infrastructure due to the amount, complexity, and sensitivity of data. SAP (SAP Software Solutions, Walldorf, Germany) [6] is an enterprise software widely used in health care organizations worldwide for data administration and processing. SAP and similar platforms require robust and complex infrastructures, which includes database management systems (DBMS). The SAP platform includes a Business/Data Warehouse (BW) module with either in-disk SQL DBMS or the more modern, highly performant in-memory HANA (SAP Software Solutions, Walldorf, Germany) database. The BW is a central platform that synchronizes and standardizes data structures from different systems. It provides input data that can be used by CDSS and algorithms to perform complex analyses using well-established techniques from statistics and computer science [7-9].

Remarkably, laboratory diagnostics are highly standardized, quality assured, and one of the most important sources for clinical decision making [10,11]. Many results are of numeric nature (eg, K^+^ [potassium] = 3.9 mmol/L), allowing for rule sets of control values to be defined by medical specialists. This, in turn, creates an ideal context for a CDSS based on programming logic. With this approach, thousands of results can be evaluated, and notifications can be sent whenever critical conditions are detected, providing valuable assistance to health care professionals. Systems that evaluate laboratory results for specific purposes of their facilities have been reviewed in [12]. To our knowledge, however, until now, there are no systems available that are capable of working with different laboratory biomarkers in parallel, are scalable, and can identify complex laboratory assemblies, apart from AMPEL (Analysis and Reporting System for the Improvement of Patient Safety through Real-Time Integration of Laboratory Findings). Alert fatigue is a recorded problem of CDSS [13,14] and should be carefully addressed. However, laboratory measurements are patient-specific and, when used in a CDSS context, show a good balance between overalerting and underalerting [15].

### Objectives

In this contribution, we first present guidelines and a theory for a reactive software agent (RSA) for implementing a CDSS that performs laboratory diagnostics (labCDSS). To offer practical support, we present a general system with its minimal components, so that other colleagues can implement a labCDSS in their own medical facilities. We also present our specific implementation and its evaluation at the University of Leipzig Medical Center (ULMC), Germany: AMPEL, which means traffic light in German.

AMPEL is essentially a notification system [16]. When the value of a laboratory parameter falls outside of predefined reference limits, a quick medical reaction is of paramount importance (eg., K^+^ ≤2.0 mmol/L, defining severe hypokalemia). The longer this medical response is delayed, the higher the risk to patient safety due to an associated risk between severe hypokalemia and sudden cardiac death [17]. The AMPEL system notifies the medical personnel when the patient’s parameters increase the chance of life-threatening conditions. Our motivation for this research project was to design, implement, and evaluate a CDSS that improves patient safety and aids health care professionals in detecting life-threatening conditions.

## Methods

### Overview

In this section, we describe the technical requirements and components for a labCDSS. With these guidelines, health information technology (IT) specialists can plan the implementation of a labCDSS at a particular medical facility. As an example, we describe an overview of the computational infrastructure of the ULMC, where we implemented AMPEL.

### Technical Requirements

Automated systems in medical facilities must satisfy certain technical requirements. Prior planning is crucial for successful implementation of a labCDSS. Afterwards, the system’s components should be constantly monitored and potentially optimized.

The following 2 sections contain (1) a collection of general CDSS requirements, which are tailored to (2) a labCDSS. For broader and legally detailed requirements, see the work by Harer and Baumgartner [18], especially chapters 2 and 5.

#### System Functionality

The main functionality of a CDSS is to deploy different types of notifications. These are sent to clinicians or to the laboratory staff who will communicate directly with them whenever a patient’s parameter is not within a safe range or a medical treatment is delayed.

#### System Performance

The performance is measured as the time a notification takes to be delivered. Delays can result from a high number of notifications or notification configurations that should be optimized. These issues influence the choices of the appropriate system components, such as database configurations, underlying hardware, and notification means (ie, socket-based, REST-interface, among others).

#### Component Failure

Component failure can also cause delays, which could ultimately affect patients. To avoid it, a working state of the CDSS should be maintained, even if some of its components fail. This is particularly challenging for a system that is partially integrated into a public infrastructure (ie, internet and power), which is subjected to planned (eg, maintenance) and unplanned (eg, wear) outages. Users should be properly informed about such eventualities.

Some components can be uncoupled from public providers (eg, using a facility-wide intranet or a separate power grid). In general, however, it is more practical to distribute them over multiple redundant components.

#### Human Error

Two types of human error are noteworthy when planning a CDSS. The first is incorrect algorithm design. To avoid this, algorithms must be precisely tailored to specific laboratory parameters or medical diagnosis as well as based on (1) medical literature, (2) retrospective statistical analysis of available data, (3) expertise from medical laboratory specialists, and (4) expertise from clinical specialists.

The second type of error can occur during the display of notifications. Notifications in the graphical user interface (GUI) must be unambiguous and descriptive. This should be carefully considered during graphical design, along with the intended level of intrusiveness in the busy environment of an inpatient unit [19].

In addition to technical requirements, medical products are also subject to clinical evaluation before they are deployed at a particular facility. The new European Union regulation 2017/745 about general safety and performance requirements in medical devices [20] requires clinical investigations to show that the medical device (1) reaches the intended purpose planned in its design and works as expected and/or (2) provides the expected clinical benefit and/or (3) shows an acceptable risk/benefit ratio, weighing the side effects against the benefits to be achieved by the device [21].

The presented labCDSS, AMPEL, complies with these safety requirements by featuring a quantifiable benefit, transparent actions, the impossibility of adverse effects, and ease of reversibility (more details in the Discussion).

### The Functional Components of a Reactive Software Agent

Agent-based architectures have been implemented for different purposes and in the medical domain [22,23]. They share 4 structural components [24]: (1) perceptive elements that receive input, (2) digitally represented domain knowledge, (3) an inference module that generates action by applying knowledge to the input, and (4) an output component that presents these actions to the patient or the attending physician. See [25] for a systematic review.

In a software agent, information continually flows into the system as well as out of it. The stream of input data is processed by the inference module using expert knowledge, which enables input interpretation and output generation.

The first medical agent architectures were designed for home care (for examples, see [26,27]; for an overview, see [28]). Currently, various agent-based software solutions have been published with the aim of improving patient care. For example, see the work by Schaaf and colleagues [29] with a system for diagnostic support in patients with rare diseases or Nguyen and colleagues [30] with a system that supports optimal antibiotic therapy.

### A Reactive Software Agent for a CDSS That Performs Laboratory Diagnostics

An RSA is comprised of 3 components, which are described in the following sections in the context of a labCDSS.

#### Input Component

**Figure 1:**
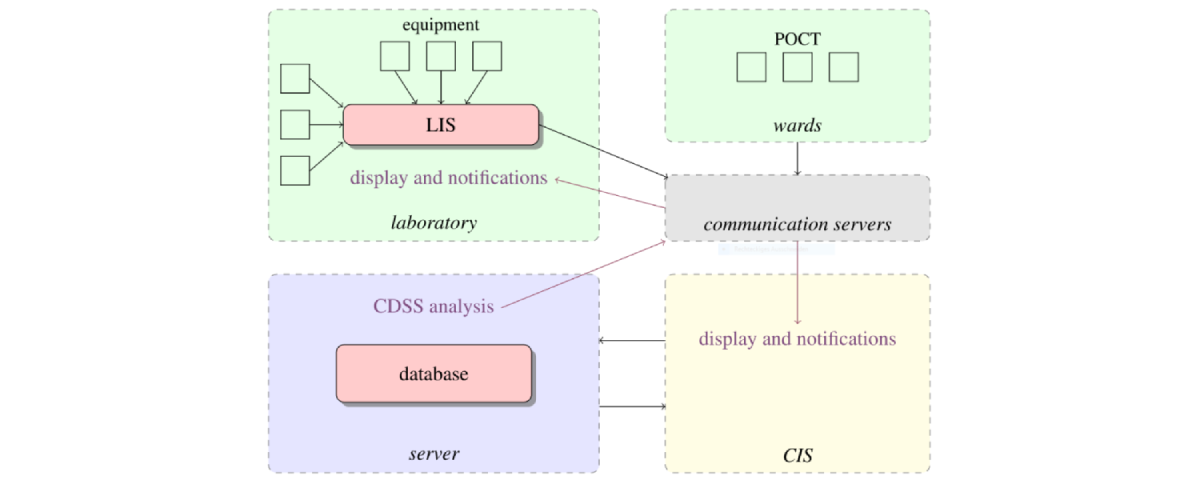
Conceptual computational infrastructure and data flow of a clinical decision support system (CDSS) that performs laboratory diagnostics following a reactive software agent framework. Input components are colored green, knowledge representation and inference components are blue, and output components are yellow. Laboratory parameters are measured by Point-of-Care Testing (POCT) devices and/or at a dedicated laboratory, stored in a Laboratory/Clinic Information System (LIS/CIS), and sent over to one or more information management systems and a server. Nodes indicate components that either generate or process data, while the edges indicate directional data transfer between the components over the internet.

The input component is depicted by green rectangles in Figure 1. The labCDSS receives input data from the laboratory information system (LIS) at a central server, which stores, manages, and processes data from various sources [31]. The input data are, however, not restricted to the LIS. The central server can also receive laboratory results over the internet, for instance.

Network packages should be encoded in standard eHealth formats, such as Health Level Seven International (HL7) or American Society for Testing and Materials (ASTM).

#### Knowledge Representation and Inference

The knowledge representation and inference is shown as the blue rectangle in Figure 1. This is the key component of the labCDSS. Although parameter evaluation could be performed in other components, an appropriate one is the central server, which often holds a data warehouse (DW) and performs extract, transform, and load (ETL) processes. The DW is the central collection point for data that are generated by various sources.

ETL processes are performed by the DW at regular intervals to keep the data up to date. ETL processes receive data from various sources, clean and unify them, and are then processed or loaded within the DW. How often data are received and formatted depends on the institutional specificities [32-34].

#### Output Component

The output components are shown in the yellow rectangle in Figure 1. The output of the labCDSS is supplied to the CIS, which is usually connected to the various systems of modern clinical facilities, such as pathology and radiology. The CIS stores information in an electronic medical record, which clinicians have access to. The format of the electronic medical record can be modified to include new data elements and entry methods. Display and special features can also be included.

### Local Computational Infrastructure

The ULMC has 6000 employees and 1451 beds with 57,000 inpatients, 374,000 outpatients, and 32,000 emergency patients annually [35]. Around 15,000 laboratory parameters are measured every day at the ULMC. Most of them are measured at the Institute for Laboratory Medicine, Clinical Chemistry and Molecular Diagnostics (ILM). The ILM is a medical laboratory that provides measurements of patients’ samples in daily routine diagnostics. It offers these services to the inpatient and outpatient units, the departments of the Faculty of Medicine, the Medical Care Center of the ULMC [36], and external institutes.

Some of the laboratory diagnostics are performed directly at the inpatient units, via point-of-care testing (POCT) devices. These include ABL90 Flex and ABL800 Flex blood-gas, metabolite, and electrolyte analyzers (Radiometer, Krefeld, Germany) [37] as well as blood glucose meters (Roche, Basel, Switzerland) [38]. After measurements, either at the ILM or directly at the inpatient units, diagnostic data is sent to the CIS and LIS via communication servers: Aqure (Radiometer, Krefeld, Germany), Kodix (in-house development), and IT-1000 (Roche, Basel, Switzerland).

SAP [6] is the CIS of the ULMC and performs several important tasks. It is not as efficient in handling laboratory results and has only limited reporting options. For these reasons, the ULMC uses LabCentre (i-Solutions health, Mannheim, Germany) [39] for this task. LabCentre is the LIS of the ULMC and is of utmost importance to the AMPEL project. It is accessed by the AMPEL team for control as well as for coordination of phone calls via an implemented phone call documentation system. This system is implemented in the LIS, so that for each algorithm or even for the whole report, telephone calls can be documented. The documentation includes the call time, phone number, and name of the called party. In parallel to LabCentre, SAP [6] contains several modules, each dedicated to a specific purpose (eg, patient care, data storage, controlling, and logistics). The SAP R/3 module has a visual interface that displays patient data and is used for patient care purposes by medical personnel. The core of AMPEL analyses occurs in the SAP BW module. The output of AMPEL is sent from the BW to the SAP R/3 module and is integrated into the inpatient unit overview in the form of a dedicated AMPEL column.

The computational infrastructure of the ULMC can be represented as a graph, in which the nodes indicate components that either generate or process data and the edges indicate directional data transfer between the components via a shared resource or socket. The BW of the ULMC is model-driven and based on the SAP NetWeaver ABAP platform [40], which runs the release version SAP NetWeaver Server Version 7 with EHP 4 for both SAP BASE and SAP BW modules.

The BW receives data from heterogeneous sources (SAP and non-SAP alike) and cleans and stores them for downstream analysis. In addition, there are 2 MSSQL databases for data storage, one connected to the BW and the other connected to SAP R/3. Communication servers transfer data between the machines that measure the laboratory parameters and the BW, CIS, and LIS. The standard format HL7 [41] is used for all data transfer, with the exception of the AMPEL system, which uses a custom format, and the transfer between Aqure and LabCentre, which uses the ASTM [42] format.

## Results

### Overview

In this section, we detail the AMPEL labCDSS as well as its implementation in the ULMC. AMPEL was planned according to the technical requirements defined in the "Technical Requirements" subsection in the Methods and implemented within the RSA framework described in "The Functional Components of a Reactive Software Agent" subsection in the Methods.

It can also be implemented in different computational infrastructures that provide the functions defined in the "A Reactive Software Agent for a CDSS that Performs Laboratory Diagnostic" subsection in the Methods.

### AMPEL at the ULMC

#### Input Component

The input to AMPEL comes from the SAP R/3 module, which, in turn, receives data from the POCT machines at the inpatient and outpatient units as well as the machines from the ILM. AMPEL is implemented in the SAP BW. Consequentially, the communication was adapted to SAP’s native data transport interfaces.

The exact content of these communication packages varies, but some core information is required: (1) a timestamp that indicates the measurement time, (2) the patient ID to associate the data with the particular medical record, (3) the case ID to relate the values to the current treatment, (4) an order ID to identify the batch of the measurements throughout the current treatment, (5) the laboratory parameter, and (6) its result.

#### Knowledge Representation and Inference

Incoming data packages are stored and processed in the SAP BW. The SAP BW is the central server of the ULMC (Figure 1, bottom left) and uses ETL processes to make the evaluations. The algorithms that compose AMPEL encode medical domain knowledge to transform the input into medically relevant output. The output of each algorithm is a classification of the patient’s parameter results regarding a particular parameter or diagnosis. Notifications are triggered whenever a value falls outside the defined range.

After processing, 1 of 3 possible outputs is generated: (1) all values are within safe ranges; (2) at least one value needs to be monitored, but no immediate action is required; or (3) at least one value is critical, and immediate action is required.

#### Output Component

Once the output has been generated, the host system converts it into an outbound message. AMPEL makes use of the secure internet communication framework (SICF) integrated into SAP and sends the resulting data as a HTTP post package to its target destination. Other communication channels (eg, REST interfaces) would also be possible.

### Implementation Details

When the laboratory results of a patient become available at the SAP BW, they are subjected to AMPEL’s algorithms. The mean time between a critical condition being detected by the algorithms and a notification being sent to the AMPEL team is 36 minutes. This time can be shorter or longer depending on how fast the data are processed throughout the computational system (for more details, refer to the Limitations section). Each algorithm is specific to a laboratory parameter or a diagnosis (eg, hypokalemia and potassium). Five algorithms have already been implemented (Table 1), and two more complex algorithms are in the prerelease phase (Table 1). In addition, up to 23 other algorithms are being developed. Importantly, each algorithm is tailored to the specificities of the parameter, diagnosis, and other relevant medical aspects. Some of them (eg, hypokalemia and hypercalcemia) have straightforward control rule sets, while others, such as creatinine, for the detection of renal failure, or procalcitonin (PCT), to diagnose and monitor infections, require more complex rule sets. Each algorithm is carefully developed by a team of physicians, scientists, and IT personnel (computer scientists, computer engineers, and bioinformaticians) under close consideration of literature and extensive practical experience. Theoretically, algorithms can be defined in any formal language that enables the description of rational functions over input values and time.

**Table 1:** Laboratory parameters, rule sets, time to control (TTC), and diagnosis from AMPEL. Each line comprises a specific algorithm. If any parameter value falls outside the defined range (rule set), the diagnosis is documented, and notifications are sent to warn clinicians in the inpatient units.

Laboratory parameter	Rule set	TTC	Diagnosis
K^+^ (potassium)	<2.5 mmol/L	≥6 hours	Hypokalemia
Ca^++^ (calcium)	>3.5 mmol/L or >2.0 mmol/L ionized	≥12 hours	Hypercalcemia
Na^+^ (sodium)	<120 mmol/L	≥12 hours	Hyponatremia
Lactate	>4 mmol/L	≥ 6 hours	Hyperlactatemia
Creatinine	Complex rule set	Immediately	Acute kidney injury
Procalcitonin (PCT)^a^	Complex rule set	Prerelease	Sepsis
Troponin T^a^	Complex rule set	Prerelease	Myocardial infarction

^a^Requires more complex rule sets and are currently under development.

Most of the algorithms were based on the following: First, the result of the laboratory parameter should indicate a critical medical finding with a high specificity. Second, the system should report if there is evidence of delayed medical interventions. Both aspects indicate the seriousness of the medical situation. For instance, a blood potassium deficiency of K^+^ ≤1.9 mmol/L is acutely life threatening and requires immediate response. Therefore, the laboratory instantly informs the inpatient and outpatient units of these cases, and adequate treatment must be initiated. If the potassium of these patients has not been checked within the last 6 hours, this critical finding may have been overlooked, and a life-threatening condition is likely to occur, due to the risk of heart rhythm disturbances.

AMPEL detects such cases computationally, and clinicians in the inpatient and outpatient units are notified to the situation via a phone call. These notifications originate at the BW, in which text messages are generated according to the algorithms. The notifications appear as items on a telephone list of the LIS, which are manually processed, either by the AMPEL team (business hours) or by a medical technologist of the ULMC (24 hours, 7 days a week).

Feedback on the system is obtained at the end of each notification call. Two questions are asked, and the answers are manually entered into the LIS: “Has the therapy started (based on laboratory findings)?” and “Do you think the notification helped in treating the patient?” This direct communication is part of the evaluation of the AMPEL system and incorporates user feedback.

**Figure 2:**
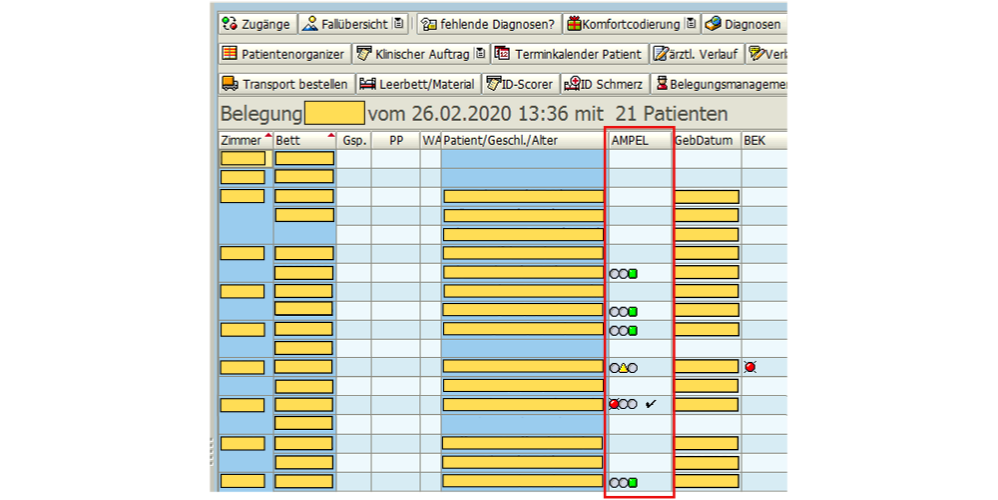
Screenshot of the SAP R/3 visual interface with highlight on the dedicated AMPEL column, with all three possible colors (summary report) displayed: green, yellow, or red.

The AMPEL displays at the SAP R/3 are assigned 1 of 3 colors: green, yellow, or red. The colors indicate the status of the evaluated parameter with regard to the health risk to the patient. As a summary of all analyzed parameters, 1 unique color will be assigned to each patient in a dedicated column at the SAP R/3 interface (see Figure 2). This assignment follows a hierarchy: If there is at least 1 red parameter, the overview color is set to red; if there are no red parameters but at least 1 yellow, the overview color is set to yellow. Green is set otherwise.

If the connection between the server and the terminals at the inpatient units fails, the AMPEL column of SAP R/3 is simply not filled with any color and remains empty. The terminal interface queries the AMPEL server every 600 seconds. As soon as a connection has been reestablished, the column appears again on the terminal.

**Figure 3:**
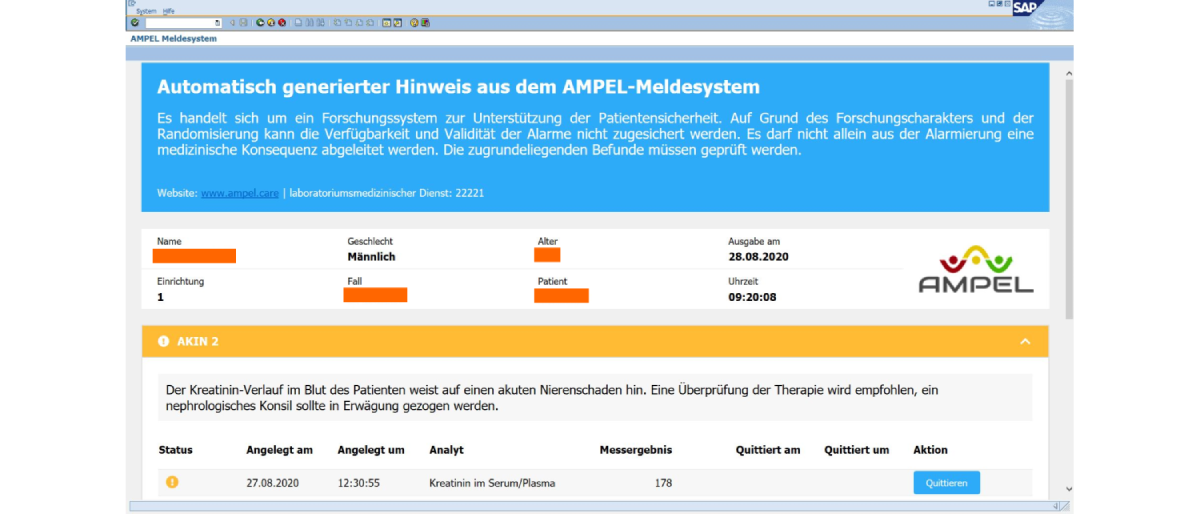
Screenshot of the interactive AMPEL report for a patient at the SAP R/3 visual interface. All algorithms that have been analyzed by the AMPEL system are displayed, each in a highlighted line. The user can access the specific reports by clicking on the highlighted line. In this example, the creatinine (AKIN 2) report for acute kidney injury has been chosen. The full report displays additional information to clinicians, such as internal standards or specifics from medical guidelines. The blue box at the top states that the analysis has been carried out automatically by AMPEL, an ongoing research project, and should therefore be used with caution.

Moreover, a user can click on a specific AMPEL symbol and get a full AMPEL report for a patient (Figure 3). This represents an overview of all the patient’s notifications and their individual assessment. Importantly, when a clinician checks all yellow and red AMPEL notifications and attends to the patient, they manually click on the checkmark in the SAP R/3 AMPEL column. The checkmark is afterwards displayed in SAP (Figure 2), informing other clinicians that the patient has already been attended to.

## Discussion

### Principal Findings

We designed and implemented the AMPEL system in the ULMC as well as the Muldental Clinics in Grimma and Wurzen in Germany. AMPEL is a labCDSS with 5 active algorithms and 2 under development. Each algorithm is specific for a laboratory parameter or a diagnosis and is carefully developed by an interdisciplinary team. If a critical condition is detected, notifications are sent to warn the patient’s attending clinicians. AMPEL is constantly being evaluated regarding functionality and usefulness.

In addition, we are currently collecting data for prospective analysis and scientific evaluation of each algorithm.

### Safety Requirements

This section is divided into 4 main categories. In extent and detail, these features of the AMPEL system address and go beyond annex I of EU regulation 2017/745 mentioned in the Technical Requirements subsection in the Methods.

#### Quantifiable Benefit

During the development phase, ULMC patients are assigned to a control or intervention group so as to quantify AMPEL’s effects. Odd patient IDs make up the control group and even IDs the intervention group. The assignment of patient IDs is incremental and nonsystematic and not expected to create a bias for any of the test groups. Both groups undergo the AMPEL analysis but only the results for the intervention group are presented in the AMPEL column of the SAP R/3 terminal (for an example, see Figure 3).

Preliminary results show that the time to control (TTC) is considerably lower for patients of the test group ([16] and unpublished data). Based on AMPEL’s transparency of actions, its deterministic algorithms, and the randomized controlled experiment setting, there is solid reason to assume this is due to AMPEL’s notifications. To further investigate these preliminary indications, trials are currently ongoing, and analysis are carried out for specific patient cohorts (eg, male versus female, patients with liver and kidney disease, and others).

#### Transparent Actions

The notifications of the system are determined by manually designed rule sets. As long as the involved components perform according to their specifications, the system successfully notifies the clinicians of all cases that are singled out by the rule set. It is important that any system action can be traced back to one unambiguous cause. This is necessary for the direction of subsequent medical attention as well as investigation in case of possible erroneous notifications. This issue is especially important to keep in mind in case the system is extended at some point — for example with a machine learning module — to guarantee the impossibility of adverse effects.

#### Impossibility of Adverse Effects

Component failure is not addressed by AMPEL as a research project because of extremely rare outages. This will become relevant, however, when AMPEL matures into a medical product. If the system is to be deployed at places with less reliable infrastructure, a coordinated distribution of its components is reasonable and should be incorporated.

The AMPEL system was designed to avoid unnecessary notifications. To achieve this, we simulate the system with retrospective patient data and evaluate the amount of less helpful notifications in coordination with clinical specialists. Furthermore, the system is continually adapted and evaluated prospectively. Up to the writing of this article, the feedback of clinicians has been very positive, although individual, less helpful notifications cannot be fully avoided. For patients in palliative medical conditions, the system can be individually silenced by the system administrator.

#### Ease of Reversibility

In the unlikely case that the system consistently generates false notifications, this could result in an unfavorable redistribution of a clinician’s attention. If this occurs, the system can be easily removed from the computational infrastructure.

AMPEL does not replace any other notification system. Therefore, problems caused by the removal of the AMPEL column (see Figure 2) might arise only if staff has started to depend on AMPEL as their sole means of a reminder.

### Limitations

Considering the complexity of the computational infrastructure and the amount of input data at the ULMC, unforeseen issues may arise in the AMPEL system, such as delays or connectivity outages. In order to localize and solve these problems, thorough documentation is required, along with continuous monitoring and communication between users and system management.

After several months of evaluation, delays were identified within the current implementation of AMPEL. The most frequent one was found within the on-disk SAP BW database itself, contrary to a faster in-memory, and is only accessed by the BW in fixed time intervals (every 60 minutes). Due to this delay, follow-up examinations might already have taken place but are still displayed as pending in the SAP R/3 AMPEL column. We are in the process of moving our system to an in-memory infrastructure to minimize this delay.

Another reason for delay has been identified in the Kodix communication server. It sends parameter values to SAP R/3 only every 15 minutes. In case of a connection break, parameters must be sent manually by the Kodix IT team. Kodix belongs to the critical IT infrastructure. Outside working hours, a computer scientist is available on call at the hospital.

In order to avoid delays of the AMPEL evaluation, we developed systematic documentation in the form of a checkbox protocol, based on the dataflow of a parameter measurement. As an example, if we notice a large time lag between the measurement of the POCT machine and the timestamp of the Aqure connecting server, we can pinpoint the delay to the connecting servers Aqure or Kodix. Alternatively, if the lag occurred between the TTC and the timestamp of the email (both being assigned at the SAP BW), we can pinpoint the delay to the internal system of AMPEL in the SAP BW.

Problems regarding the components of the computational infrastructure are inherited by the AMPEL system. If one of the communication servers is under maintenance, messages to the LIS can be sent twice. In our settings, unscheduled suspension of the Kodix server, for example, leads to a resubmission of messages from the previous day, causing duplications.

Another issue was detected with overlooked or misjudged notifications at the LIS interface (on which phone calls are based). Part of this can be avoided simply by reducing delays in message delivery, such that there is no ambiguity as to the notification’s current relevance and whether it should still be considered.

The data of the ULMC are mirrored in the SAP BW server. This ensures availability of data and provides a redundant means of storage. Conservative default settings set the maximum query frequency for the secondary database to 1 hour. In some cases, this can lead to notifications arriving after the necessary treatment has been initiated. Given the proper functionality of all system components, the maximum delay between measurement and notification is 85 minutes (up to 15 minutes for the Kodix server to send data, up to 60 minutes for the SAP BW to access the on-disk database, and up to 10 minutes for the inpatient terminal display to update.)

### Conclusions

A labCDSS can be used to notify clinicians of important follow-up procedures. It interprets and processes laboratory data at a central node and delivers the results to a team who notifies the attending clinicians.

An overwhelming amount of digitally recorded medical data is available in the CIS of a medical facility, which can yield valuable information, but is impossible to analyze manually. The rule sets of the AMPEL system are developed and verified by laboratory medicine specialists under critical consideration of current medical literature. These rules are implemented in the algorithms of AMPEL, which automatically process the large amount of available laboratory parameters and discover associations between the parameter results and diseases.

AMPEL and similar labCDSS could be a valuable asset in clinics in remote areas as well as smaller facilities that do not have in-house laboratory medicine experts, dedicated to screen laboratory results for improved patient safety. We presented in this contribution the technical requirements and functional components of a CDSS for laboratory diagnostics and exemplified them by detailing our implementation of the AMPEL system at the ULMC. We hope to encourage colleagues to also design and implement a labCDSS within their institutions.

## Abbreviations

AMPEL: “Analyse- und Meldesystem zur Verbesserung der Patientensicherheit durch Echtzeitintegration von Laborbefunden,” which translates to “Analysis and Reporting System for the Improvement of Patient Safety through Real-Time Integration of Laboratory Findings”
ASTM: American Society for Testing and Materials
BW: Business Warehouse
CDSS: clinical decision support system
CIS: clinical information system
DBMS: database management system
DW: data warehouse
ETL: extract, transform, and load
GUI: graphical user interface
HL7: Health Level Seven International
ILM: Institute for Laboratory Medicine, Clinical Chemistry and Molecular Diagnostics
IT: information technology
K: potassium
labCDSS: CDSS that performs laboratory diagnostics
LIS: laboratory information system
PCT: procalcitonin
POCT: point-of-care testing
RSA: reactive software agent
SICF: secure internet communication framework
TTC: time to control
ULMC: University of Leipzig Medical Center
